# High-Fat Diets Disturb Rat Epididymal Sperm Maturation

**DOI:** 10.3390/ijms26051850

**Published:** 2025-02-21

**Authors:** Lorena Ruiz-Valderrama, José Edwin Mendoza-Sánchez, Ernesto Rodríguez-Tobón, Isabel Arrieta-Cruz, Humberto González-Márquez, Pablo Arturo Salame-Méndez, Rosario Tarragó-Castellanos, Edith Cortés-Barberena, Ahiezer Rodríguez-Tobón, Edith Arenas-Ríos

**Affiliations:** 1 Departamento de Biología de la Reproducción, Universidad Autónoma Metropolitana, Iztapalapa, Ciudad de México 09340, Mexico; anerol85@xanum.uam.mx (L.R.-V.);; 2 Doctorado en Biología Experimental, Universidad Autónoma Metropolitana, Iztapalapa, Ciudad de México 09340, Mexico; 3 Departamento de Investigación Básica, Instituto Nacional de Geriatría, Magdalena Contreras, Ciudad de México 10200, Mexico; 4 Departamento de Ciencias de la Salud, Universidad Autónoma Metropolitana, Iztapalapa, Ciudad de México 09340, Mexico

**Keywords:** overweight, obesity, infertility, epididymis, sperm parameters

## Abstract

Infertility is increasingly recognized as being closely linked to obesity in humans. The successful production of fertile spermatozoa requires adequate spermatogenesis within the testis and proper spermatozoa maturation through the epididymis. This study aimed to evaluate the impact of body adiposity on male fertility, focusing on sperm parameters, epididymal sperm maturation, and sperm capacitation in Wistar rats. Male rats were randomized into three dietary groups over four weeks: a control group receiving less than 4% lard, regular chow, a 10% lard group, and a 60% lard group. Following dietary interventions, fertility tests were conducted across the groups. The epididymis was dissected into caput, corpus, and cauda regions to assess sperm concentration, vitality capacitation, carbohydrate distribution, tyrosine phosphorylation, and phosphatidylserine levels. Additionally, serum testosterone levels were measured to evaluate hormonal influences on fertility. The rats subjected to high-fat diets leading to overweight and obesity exhibited significant alterations in fertility. These changes were characterized by impaired epididymal sperm maturation, as evidenced by lower testosterone levels, decreased sperm viability, and capacitation. Furthermore, increased adiposity was associated with a lack of asymmetry in the plasma membrane, alteration in carbohydrate distribution, and changes in tyrosine phosphorylation. This study underscores the adverse effects of high-fat diets on male fertility, particularly through mechanisms affecting sperm maturation in the epididymis. The evidence suggests that obesity-induced alterations in sperm parameters and hormonal profiles may contribute to reduced fertility in male rats, which could have implications for understanding similar human processes.

## 1. Introduction

Obesity is a complex, multifactorial metabolic disorder that affects millions of individuals worldwide. It is characterized by an abnormal accumulation of adipose tissue, which is often accompanied by a significant inflammatory component that contributes to the development of various comorbidities, including cardiovascular diseases, type 2 diabetes, and certain cancers [1,2]. Recent studies have indicated a concerning association between obesity in men and infertility, highlighting the need for further investigation into the underlying mechanisms [3,4]. The proper production of fertile spermatozoa necessitates the optimal functionality of Sertoli and Leydig cells, which are essential for steroidogenesis and spermatogenesis through the hypothalamic–pituitary–testicular axis (HPTA). However, if sperm are extracted from the testis, they are not capable of fertilizing [5].

In this context, the epididymis plays a crucial role as an androgen-dependent organ, comprising a tightly coiled tube that is anatomically divided into three main regions: caput, corpus, and cauda [6]. During their transit through the epididymis, spermatozoa undergo a series of biochemical and physiological changes that are critical for acquiring motility. These series of transformations are collectively referred to as epididymal sperm maturation [7].

Epididymal sperm maturation encompasses several modifications, including alterations in the composition of phospholipids, cholesterol, and membrane proteins, as well as changes in the phosphorylation of tyrosine residues and the redistribution of membrane carbohydrates such as N-acetylglucosamine (N-AG), sialic acid, fucose, and mannose [8,9,10,11]. These modifications are essential for spermatozoa to recognize and bind to the oocyte, acquire the ability to undergo capacitation and the acrosomal reaction and ultimately achieve fertilization [12]. Numerous studies have documented the detrimental effects of obesity on human spermatozoa, revealing alterations in sperm parameters that may compromise fertility [13,14]. In rodent models, an obesogenic environment has been shown to reduce daily sperm production, decrease testosterone levels, increase estradiol and leptin levels, and elevate lipid peroxidation within the epididymis, leading to the formation of apoptotic bodies in the epithelial tissue [15,16]. Despite these findings, there remains a paucity of information regarding the specific alterations in sperm parameters in rats exhibiting increased adiposity. Therefore, the primary aim of this study was to evaluate the effects of diet-induced obesity on sperm maturation within the rat epididymis.

## 2. Results

### 2.1. Weight Gain

**Figure 1:**
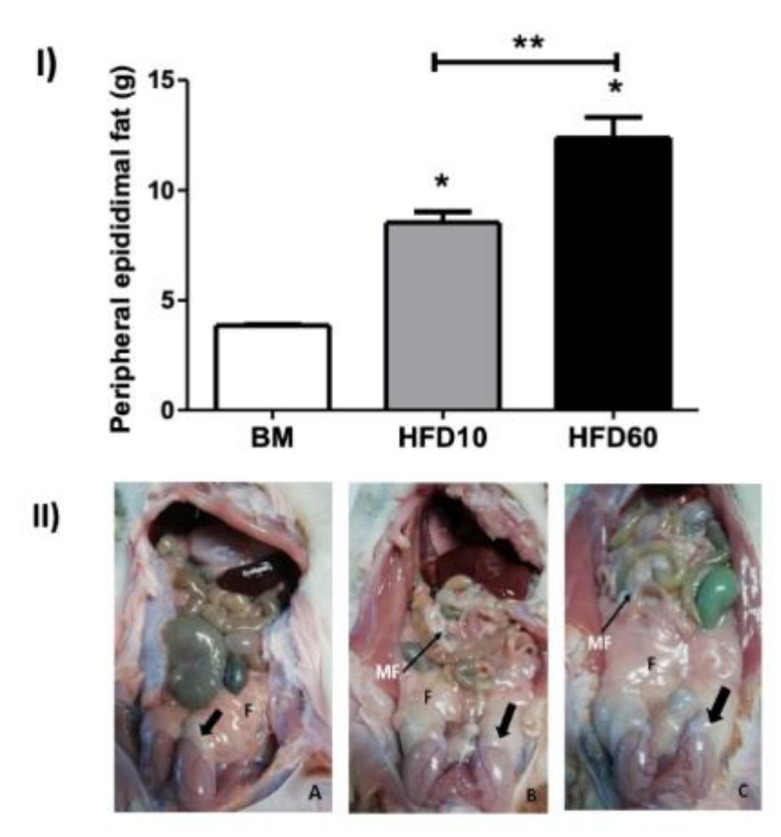
Distribution and weight of fat deposits in Wistar rats under varying dietary conditions. (**I**) Quantitative analysis of epididymal fat weight: balanced meal, high-fat diet (10%), and high-fat diet (60%) (*n* = 6). (**II**) Morphological assessment of fat distribution, highlighting total fat (F), mesenteric fat (MF; indicated by the slim black arrow), and peripheral epididymal fat (indicated by the gross black arrow) in Wistar rats across three dietary conditions: balanced meal (**A**), high-fat diet 10% (**B**), and high-fat diet 60% (**C**). * Represents significant differences between the experimental group and the control group. ** Represents *p* < 0.05.

The analysis of body weight in Wistar rats revealed that those subjected to a high-fat diet exhibited a significant increase in body weight compared to the control group. Specifically, the group receiving a diet consisting of 10% lard had a 19% increase in body weight, while the group receiving a diet with 60% showed a more pronounced increase of 34% relative to the control group (Table 1). Furthermore, the assessment of epididymal fat deposits indicated substantial differences between the dietary groups. The 10% lard group exhibited a 221% increase in epididymal fat with an average weight of 8.54 g ± 1.20 compared to the control group with an average epididymal fat weight of 3.85 g ± 0.07). In the 60% lard group, epididymal fat was even more pronounced, showing 321% with an average weight of 12.38 g ± 2.30 compared to the control group (Figure 1(I)). Notably, fat accumulation in the 60% lard group was so extensive that it covered the head of the epididymis (Figure 1(II)).

### 2.2. Testosterone Levels

**Figure 2:**
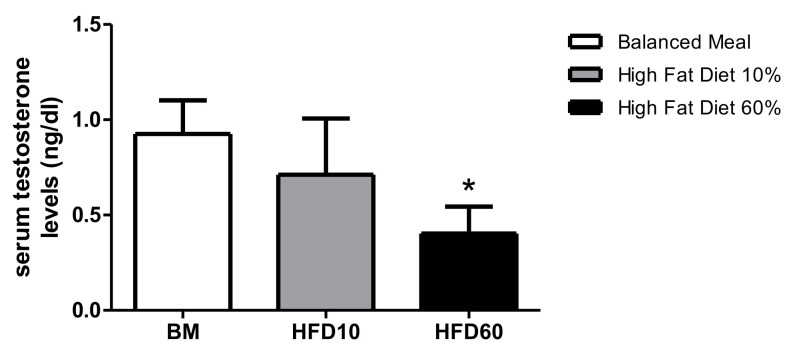
Serum testosterone levels in Wistar rats Under different dietary regimens: balanced meal, high-fat diet (10%), and high-fat diet (60%). Values (*n* = 6) are expressed as mean ± standard deviation (SD). Statistical analysis indicates a significant difference between the balanced meal and the high-fat diet 60% groups (*p* < 0.05). * Represents significant differences between the experimental group and the control group.

The testosterone serum levels between the overweight and control groups (0.712 ng/dL ± 0.295 vs. 0.926 ng/dL ± 0.176) were lower respectively. However, testosterone serum levels in the obese group (0.546 ng/dL ± 0.202) showed a significant decrease compared to the control group (Figure 2).

### 2.3. Sperm Parameters

Sperm parameters, such as concentration and viability, were measured from different sections of the epididymis; significant changes were not observed in the sperm concentration between caput, corpus, and cauda sections among the groups. Sperm viability was significantly decreased in the caput of the 10% lard group compared to the control and 60% lard group. Sperm concentration was also diminished in the corpus section of the 60% lard group compared to the other groups (Table 1).

### 2.4. Sperm Capacitation

**Figure 3:**
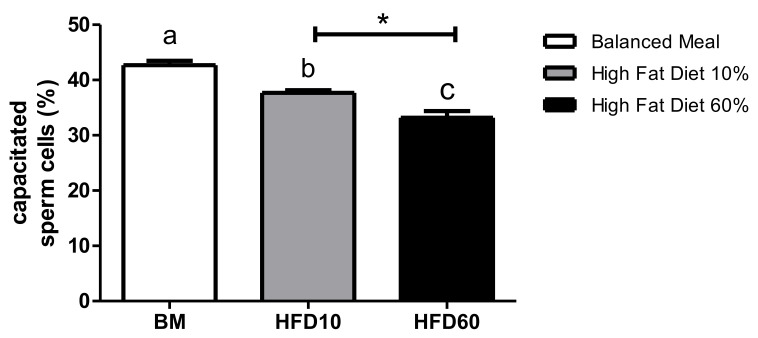
Capacitated sperm cells, as determined by the chlortetracycline (CTC) assay, after a 6-h incubation period in a capacitation medium. Data (*n* = 6) are expressed as average ± standard deviation (SD). Different letters (a, b, c) indicate significant differences. Specifically, a significant difference was observed between the high-fat diet 10% (HFD10) and high-fat diet 60% (HFD60) groups (*p* < 0.05). * Represents significant differences between the experimental group and the control group.

Dates showed a significant decrease in the 10% lard group (11.7%) and the 60% lard group (23.3%) compared with the control group and a significant difference between the 10 and 60% lard groups (Figure 3).

### 2.5. Translocation of Phosphatidylserine

**Figure 4:**
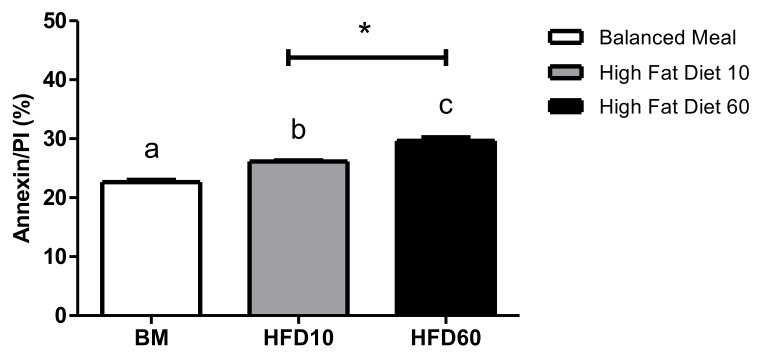
Phosphatidylserine externalization in sperm cells from high-fat diet Wistar rats, presented as average ± SD. Different letters (a, b, c) indicate significant differences. Specifically, a statistically significant difference was observed between the high-fat diet 10% (HFD10) and high-fat diet 60% (HFD60) groups (*p* < 0.05) (*n* = 6). * Represents significant differences between the experimental group and the control group.

Residues to the outer layer of the spermatozoon plasma membrane in the cauda region of the epididymis showed a significant increase in the 10% lard group (13%) and 60% group (30%) relative to the control group and a difference between the latter (Figure 4).

### 2.6. Carbohydrate Distribution

The carbohydrate distribution in the sperm membrane was determined by counting the number of sperm cells that present different staining patterns, finding two patterns: dyed head and total staining.

### 2.7. N-Acetyl-glucosamine and Sialic Acid

The sperm N-acetyl-glucosamine and sialic acid were observed from different sections of the epididymis. The sperm-dyed head was significantly lower in the caput of both the 10% and 60% lard groups compared to the control group. Although, the total sperm stained was significantly higher than the 60% lard group. The sperm-dyed head was significantly lower in the corpus of both the 10% and 60% lard groups compared to the control group; however, it was significantly higher than the 60% lard group compared to the 10% lard group. Although the total sperm stain was significantly higher in both the 10% and 60% lard groups compared to the control group, it was significantly higher in the 60% lard group compared to the 10% lard group (Table 2).

### 2.8. Mannose

The sperm mannose was observed from different sections of the epididymis. However, although we found the two patterns in both groups, we did not find significant differences (Table 3).

### 2.9. Fucose

Spermatozoa obtained from different regions of the epididymis were also labeled against fucose. However, they were not observed through fluorescence microscopy, so no results are presented.

### 2.10. Carbohydrate Concentration

The concentration of carbohydrates was also determined by flow cytometry. The sperm N-acetyl-glucosamine and sialic acid were observed from different sections of the epididymis. We found that those carbohydrates were significantly lower in the caput of both the 10% and 60% lard groups compared to the control group.

The sperm mannose was observed in different sections of the epididymis. We found that these carbohydrates were significantly lower in the caput and cauda of the 60% lard group compared to the 10% lard group.

The sperm fucose was observed in different sections of the epididymis. We found that these carbohydrates were significantly lower in the caput of both the 10% and 60% lard groups compared to the control group (Table 4).

### 2.11. Tyrosine Phosphorylation

The percentage of protein sperm tyrosine phosphorylation obtained from the different regions of the epididymis was determined, finding a clear decrease in tyrosine phosphorylation when the sperm cells pass through the epididymal corpus in the control group and an increase in epididymal cauda. However, in the 10% and 60% lard groups, we did not find a significant difference (Table 5).

This is because the sperm cells in the corpus region, in both the 10% and 60% lard groups, are more phosphorylated, with a significant increase in the 60% lard group.

## 3. Discussion

Recent decades have witnessed a notable decline in sperm quality among humans, which has been closely linked to reproductive dysfunction [17], This trend underscores the multifaceted nature of obesity as a disorder that contributes to various health issues, including reproductive impairments [18]. Western diets, such as those in our country, are high in unsaturated, monounsaturated, and saturated fats. That is why the diet proposed in this study was chosen and applied for the time already indicated in other studies (30 days) where the only aim is to induce this body condition, leaving out other pathologies, such as metabolic syndrome or type 2 diabetes mellitus. Also, in this period, we can observe the effect it has on epididymal sperm maturation that occurs between 8 and 10 days in Wistar rats [19,20]. The mechanisms underlying the observed decrease in sperm quality and the associated reproductive dysfunction have been the subject of extensive investigation, mainly through molecular studies that elucidate the roles of specific enzymes and pathways.

Before the findings reported by Ruiz et al. [21], studies in overweight and obesity models hypothesized that these high-fat diets have shown, when analyzing fertility, that obesity can reduce the number of offspring per litter and, in the overweight condition, the gender distribution tends to decrease the number of males per litter, and this may be related to that fact that the sperm is highly sensitive to reactive oxygen species (ROS) in the testicles and epididymis, creating an oxidative stress environment that can damage sperm DNA. However, the same study showed that as ROS levels increase, there is also an increase in the activity of antioxidant enzymes such as superoxide dismutase, glutathione peroxidase, and catalase. These enzymes counteract the oxidative stress environment, reducing ROS levels and minimizing DNA damage. However, this does not alleviate the negative effects on hormonal aspects and reproductive dysfunction due to overweight and obesity. Teerds et al. explain that the increase in adipose tissue favors the release of some adipokines if we understand that this tissue functions as an endocrine organ. Adipocytes secrete leptin and o-estradiol; however, the more adipocytes there are, there is more secretion of these adipokines, generating a saturation of the receptors called leptin resistance. Leptin and o-estradiol impair the KISS gene, which in turn alters the functioning of the hypothalamus–pituitary–gonadal axis [22]. The negative hormonal effects observed by Ruiz et al. [21] align with the present study’s findings, which show decreased testosterone levels (Figure 2), affecting epididymal functionality as determined by various indicators of epididymal sperm maturation.

The epididymal sperm maturation process is critically dependent on several factors, such as hormonal particularly of dihydrotestosterone, which has its receptor in the main cells of the epididymal epithelium, and its action in the nucleus of this same cell promotes protein synthesis, sperm concentration, and the biochemical composition of sperm membranes [23]. Notably, phospholipids, cholesterol, and membrane protein content decrease as spermatozoa transit through the epididymis. A detailed analysis of membrane phospholipids revealed that approximately 44% consist of phosphatidylserine, sphingomyelin, and di-phosphatidylglycerol. Previous research by Hall et al. [24] indicated that the molar ratio of phosphatidylserine increased as spermatozoa moved from the caput to the corpus of the epididymis. These findings suggest that the fluidity and biochemical composition of the sperm membrane undergo significant changes during epididymal maturation. The current study highlights the detrimental effects of high-fat diets on sperm parameters and epididymal sperm maturation, including capacitation, carbohydrate distribution, and tyrosine phosphorylation in rat epididymal sperm.

Additionally, there was a notable translocation of phosphatidylserine residues to the outer layer of the sperm plasma membrane. These observations suggest that increased adiposity (Figure 1) disrupts the normal process of epididymal sperm maturation. Excessive adiposity correlates with alterations in the body mass index, Lee index, and leptin serum levels in rodent models [25,26,27,28]. A body mass index increase of 15% or more is considered overweight, while a 20% increase indicates obesity [26,27,29,30,31,32,33,34,35,36]. Interestingly, obese animals showed lower serum testosterone levels alongside significant epididymal fat accumulation. This phenomenon may be attributed to adipose tissue deposits surrounding the epididymis, which can enhance the aromatization of androgens to estrogens in the testis, affecting spermatogenesis [19,37,38,39].

Chronic exposure to a high-fat diet has been associated with DNA damage and reduced sperm capacitation, potentially linked to increased secretion of pro-inflammatory cytokines such as interleukin 6 (IL-6), tumor necrosis factor-alpha (TNF-α), adiponectin, and resistin. These molecules have been detected in the testes and epididymis of obese rats [40,41,42]. Sertoli and Leydig contribute to this inflammatory state through cytokine production, which can disrupt the hypothalamic–pituitary–testicular axis, leading to reduced testosterone levels [42,43]. This reduction in testosterone not only impairs capacitation but also contributes to DNA damage and alteration in the asymmetry of the sperm plasma membrane 30, which is consistent with phosphatidylserine observed in this study (Figure 4).

Tyrosine residue phosphorylation is a critical process for the activation or inhibition of proteins involved in epididymal sperm maturation. For example, transition protein 2 (TP2), expressed during specific stages of spermiogenesis, undergoes phosphorylation immediately after synthesis. The specific isoform of the subunit of protein kinase A has been identified as key to the phosphorylation of these transition proteins. The difference in tyrosine phosphorylation observed as spermatozoa transit from the caput to the corpus and cauda region in control rats was not evident in overweight and obese rats (Table 4). During spermiogenesis, the acrosome differentiates from the Golgi apparatus, which is rich in sialic acid and sulfated carbohydrates) [44]. Previous studies have identified the N-acetylglucosamine (NacG-AcS) binding site in the acrosome region distributed throughout the sperm as it passes through the epididymis [45,46].

NacG-AcS can play a role in disabling factors [47] and in oocyte recognition and binding, like fucose [48,49,50], which is present in higher concentrations in spermatozoa from the caput than those obtained from the corpus and cauda [45]. In our present work (Table 2), we observed changes in the distribution of NacG-AcS/sialic acid from the moment spermatozoa enter the caput region of the epididymis, with these differences persisting in the corpus region among the balanced meal, high-fat diet 10%, and high-fat diet 60% groups. Although fucose was not detected via fluorescence microscopy, concentration analysis revealed changes in sperm obtained from the caput region of the epididymis.

Similarly, while no differences in the distribution of mannose residues were found, a significant decrease in concentration was observed in sperm from the caudal region. Mannose is primarily localized in the acrosome, and its distribution may alter as spermatozoa traverse the epididymis. Given the critical role of these carbohydrates in sperm maturation processes, changes in their distribution or concentration could significantly impact epididymal sperm maturation and, consequently, male fertility.

## 4. Materials and Methods

All experiments were performed in compliance with the Mexican Law for Animal Handling and Protection Guidelines (NOM.062.ZOO 1999). The rats were obtained from the animal vivarium of the Metropolitan Autonomous University, Iztapalapa Campus, Mexico City. The experimental protocol was approved by the Institutional Animal Care and Use Committee.

### 4.1. Animals

This study was performed in 3-month-old male Wistar rats. The animals were individually caged under controlled light conditions (lights on from 18:00 to 6:00 h; inverted cycle) with ad libitum access to either regular chow or a high-fat diet and tap water. Animals (*n* = 6 rats per group) were randomly assigned to three different groups: (1) the control group with regular chow and 4.5% lard (protein 24.1%, fat 6.4%, sucrose 3.25%, starch 21.9%); (2) the 10% lard (protein 24.8%, cholesterol, 27 ppm, linoleic acid 2.38%, arachidonic acid 0.01%, Omega-3 fatty acids 0.29%, total saturated fatty acids 1.83%, total monounsaturated fatty acids 2.03%, polyunsaturated fatty acids 2.38%, carbohydrates 51.3%) group; and (3) the 60% lard (protein 22.6%, cholesterol, 301 ppm, linoleic acid 5.09%, arachidonic acid 0.06%, Omega-3 fatty acids 0.39%, total saturated fatty acids 13.79%, total monounsaturated fatty acids 14.09%, polyunsaturated fatty acids 5.15%, carbohydrates 25.9%) group (LabDiet, St. Louis, MO, USA). The animals had free access to each diet for four weeks. Body weight was measured daily at the same time.

The animals were sacrificed and examined post-mortem to determine the weight of the epididymal fat depot. Blood samples were collected and centrifuged at 1500× *g* for 15 min. The serum was kept at −20 °C until processing. The epididymal fat from the right and left testicles was removed and weighted, and, subsequently, the epididymis was split into three sections (caput, corpus, and cauda) and the sperm was squeezed out from each section to evaluate its motility, concentration, vitality, and capacitation. DNA damage, the lack of asymmetry in the plasmatic membrane, and ROS were also assessed.

### 4.2. Testosterone Serum Levels

ELISA used a commercial testosterone kit (CAT NO. EIA 1559 DRG Instruments Inc Frauenberg, Marburg, Germany), following the instructions in the manufacturer’s manual.

### 4.3. Sperm Parameters

Each section of the epididymis was rinsed with Ringer physiological solution (NaCl 95 mM, KCl 5 mM, CaCl_2_ 1.7 mM, KH_2_PO_4_) at 37 °C and squeezed out with small scissors to remove the spermatozoa. A total of 10 µL of the solution was placed on a sliding glass stained with eosin–nigrosin for 1 min at 37 °C, and approximately 100 cells were counted under an optical microscope with a 40× objective (Optisum DESEGO, Narvarte, CDMX). Sperm concentration (millions/mL) was determined using a Neubauer chamber (LAB COMERCIAL, Barcelona, Spain) [1].

### 4.4. Sperm Capacitation

Spermatozoa from the epididymal cauda were placed in capacitation medium (94.6 NaCl, 25 mM KCl, 1.71 mM CaCl_2_, 1.19 mM MgSO_4_, 1.19 mM KH_2_PO_4_, 25 mM NaHCO_3_, 5.56 mM glucose, 10.76 mM sodium lactate, 0.5 mM sodium pyruvate, and 4 mg/mL bovine serum albumin, pH 7.4) and were incubated for 6 h at 37 °C plus 5% CO_2_. After incubation, the sample was fixed with 4% paraformaldehyde. A total of 15 µL of CTC solution (20 mM de Tris, 130 mM de NaCl, 5 mM cysteine, and 1.5 mM CTC, pH 7.8) and DABCO (220 mM DABCO was dissolved in PBS and glycerol in a proportion of 9:1) at a 1:1 ratio (15 µL) were added. The sample was examined with an epifluorescence microscope with 405 nm excitement, 460 nm fluorescence, and 510 nm filter (Olympus BX41; JIP Inc., Tokyo, Japan) with 100× objective. Approximately 100 cells were counted. Grading of the fluorescence patterns was performed as follows: (A) a non-capacitated spermatozoa pattern, (B) a capacitated spermatozoa pattern with a dark band in the post acrosomal region, and (C) an acrosome reaction pattern with weak fluorescence in the head [50].

### 4.5. Annexin V Staining

To detect surface exposure of endogenous phosphatidylserine, the FITC labeled Annexin-V-FLUOS was used (Roche Applied Science, Mannheim, Germany). After spermatozoa were induced to capacitation, they were incubated with 1 mg/mL of Annexin V-Fluos for 15 min at 37 °C with 5% CO_2_ and then closed in the dark. Subsequently, the sample was examined with an epifluorescence microscope (488 nm) with a 100× objective; approximately 100 cells were counted [51].

### 4.6. Carbohydrate Distribution

Identification of sperm membrane carbohydrates was performed with FITC-conjugated lectins (Sigma, St. Louis, MO, USA) as Fierro et al. described [48]. *Triticum vulgaris* agglutinin (WGA), *Canavalia ensiformis* agglutinin (Con-A), and *Ulex europaeus* agglutinin (UEA) were used to identify sialic acid and/or N-acetylglucosamine, D-mannose, and L-fucose. Approximately 3 × 106 sperm cells were incubated with 15 µL of lectin previously diluted to 1:50 in phosphate-buffered saline (PBS) for 30 min at 36 °C. They were washed twice and centrifuged at 500× *g* with Ringer’s solution. The pellet of cells was fixed in 1% paraformaldehyde for 1 h at room temperature and was washed with PBS before analysis.

An aliquot was analyzed with an epifluorescence microscope (Optisum DESEGO, Narvarte, Ciudad de México, Mexico) using a specific filter for FITC (460 nm excitation, 490 nm fluorescence, and 520 nm barrier filter). One hundred spermatozoa were counted and classified according to identified patterns. Controls were prepared by incubating the lectins with their target.

A sample of one million cells incubated with lectin was analyzed in a flow cytometer model FACSCalibur (BD Bioscences, San José, CA, USA). At least 10,000 cells were collected by sample and analyzed using Flowing software version 2.5.1. The fluorescence index was obtained from fluorescence intensity histograms compared with the number of marked cells. A control sample was prepared by preincubating the lectin with the corresponding carbohydrate at a concentration of 0.3 M for 30 min and subsequently adding one million cells [45,48].

### 4.7. Tyrosine Phosphorylation

An aliquot of 1 × 10^6^ sperm cells/mL was incubated with FITC-conjugated anti-phosphoprotein tyrosine monoclonal antibody (2 μg/mL) (clone Py-20-FITC, Sigma Chemical Co., St. Louis, MO, USA) in PBS containing 0.1% BSA for 5 min at room temperature. A non-specific antibody (IgG2b, Sigma Chemical Co., St. Louis, MO, USA) was used as a control to determine non-specific binding. Labeled samples were analyzed using a FACSCalibur (BD Bioscences, San José, CA, USA). For flow cytometric analysis, 1 × 10^6^ sperm aliquots were taken, and 20,000 cells per sample were evaluated [8].

### 4.8. Statistical Analyses

The data are presented as average ± standard deviation and were analyzed with an Omnibus Normality and Modified Levene Equal Variance Test and one-way analysis of variance (ANOVA). Parametric Tukey–Kramer or non-parametric Kruskal–Wallis tests were used with their respective post hoc tests. A probability value of *p* < 0.05 was considered statistically significant. The statistical analyses were performed using NCSS 2007 DATA Software, version 1, Inc. (Kaysville, UT, USA).

## 5. Conclusions

In summary, this study provides compelling evidence that high-fat diets induce significant alterations in male reproductive health through mechanisms involving hormonal disruption, oxidative stress, and changes in sperm maturation processes. These findings highlight the importance of dietary management in addressing obesity-related reproductive themes and underscore the need for further research into molecular issues to explore potential therapeutic interventions aimed at mitigating the adverse effects of obesity on male fertility.

## Figures and Tables

**Table 1:** Epididymal sperm parameters of Wistar rats fed high-calorie diets (average ± SD, *n* = 6).

	Sperm Parameters					
	Viability (%)			Concentration (millions/mL)		
	Caput	Corpus	Cauda	Caput	Corpus	Cauda
BM	53.2 ± 6.112	34.6 ± 13.410	52.8 ± 19.903	80.75 ± 35.08	47 ± 26.589	112.5 ± 41.889
HFD10	20.8 ± 20.507 *	15.9 ± 15.951	35.5 ± 22.378	101.07 ± 52.093	27.5 ± 19.780	124.85 ± 65.235
HFD60	38.8 ± 8.376	24.6 ± 21.190	41.2 ± 25.972	112.5 ± 55.396	10.1 ± 6.045 *	100.5 ± 41.889

* Represents significant differences between the experimental group and the control group.

**Table 2:** Distribution of N-acetyl-glucosamine and sialic acid in the epididymal spermatozoa of Wistar rats fed with hypercaloric diets (average ± SD, *n* = 6).

Groups	Caput		Corpus		Cauda	
	Dyed Head	Total Staining	Dyed Head	Total Staining	Dyed Head	Total Staining
BM	63 ± 5 a	27 ± 2 a	69.5 ± 10.5 a	25.5 ± 10.5 a	74.5 ± 1.5 a	21 ± 1 a
HFD10	31.67 ± 7.64 b	54.33 ± 7.77 b	31 ± 3.61 b	60.67 ± 1.15 b	44.6 ± 29.3 a	36 ± 18.5 a
HFD60	30 ± 2 c	55 ± 10 c	33.5 ± 3.5 c	55.5 ± 2.5 c	24.6 ± 22.8 a	24.6 ± 22.8 a

Different letters indicate statistically significant differences (BM vs. HFD10 vs. HFD60. *p* < 0.05).

**Table 3:** Mannose distribution in the epididymal spermatozoa of Wistar rats fed high-calorie diets (average ± SD, *n* = 6).

Groups	Caput		Corpus		Cauda	
	Dyed Head	Total Staining	Dyed Head	Total Staining	Dyed Head	Total Staining
BM	77.5 ± 0.5	6.5 ± 0.5	74.5 ± 0.5	14.5 ± 0.5	76 ± 6	14.5 ± 8.5
HFD10	70 ± 24.5	13.6 ± 8	45.6 ± 36	29 ± 28.7	51 ± 36.6	34 ± 25.6
HFD60	67 ± 19.6	15.3 ± 8	67 ± 24	16 ± 12.6	60.6 ± 34	28.3 ± 25

**Table 4:** Fluorescence intensity from WGA, ConA, and UEA in epididymal sperm (average ± SD, *n* = 6).

Composition of Glycoproteins in Spermatozoa						
Carbohydrates	BM		HFD10		HFD60	
N-acetyl-glucosamine and sialic acid	caput	279.7 ± 26.87	caput	309.3 ± 113.4 **	caput	118.2 ± 73.69 *
	corpus	147.1 ± 108.4	corpus	387.8 ± 209.4	corpus	208.5 ± 35.79
	cauda	141.14 ± 91.84	cauda	148.5 ± 3.55	cauda	83.82 ± 50.23
Mannose	caput	329.2 ± 117.6	caput	568.4 ± 334.6	caput	98.97 ± 5.46 **
	corpus	545 ± 400.5	corpus	337.6 ± 280.6	corpus	212.6 ± 96.64
	cauda	85.07 ± 36.93	cauda	130.7 ± 59.93	cauda	56.18 ± 14.01 **
Fucose	caput	8.78 ± 6.07	caput	120.8 ± 101.6 *	caput	5.31 ± 4.6 **
	corpus	13.08 ± 2.9	corpus	62.13 ± 43.71	corpus	16.65 ± 10.41
	cauda	3.7 ± 0.7	cauda	30.82 ± 21.1	cauda	6.6. ± 1.5

* BM vs. HFD60, ** HFD10 vs. HFD60. *p* < 0.05.

**Table 5:** Tyrosine phosphorylation (%) in Wistar rat spermatozoa. Wistar rats were fed a balanced diet (BM) and 10% and 60% fat food (HFD10 and HFD60, respectively) (average ± SD, *n* = 3).

Groups	Epididymis Regions		
	Caput	Corpus	Cauda
BM	14.84 ± 4.1	2.05 ± 0.7 a *	3.95 ± 2.9 *
HFD10	7.02 ± 2.9	4.15 ± 3.9 a	3.53 ± 1.8
HFD60	6.45 ± 3.2	11.06 ± 3.6 b	6.13 ± 2.9

Different letters indicate significant differences (BM vs. HFD10 vs. HFD60). * Indicates significant differences (caput vs. corpus vs. cauda).

## Data Availability

The dataset is available upon request from the authors.
